# Screening of Autophagy-Related Prognostic Genes in Metastatic Skin Melanoma

**DOI:** 10.1155/2022/8556593

**Published:** 2022-01-13

**Authors:** Cao-Jie Chen, Hiroki Kajita, Noriko Aramaki-Hattori, Shigeki Sakai, Kazuo Kishi

**Affiliations:** Department of Plastic and Reconstructive Surgery, Keio University School of Medicine, Tokyo 160-8582, Japan

## Abstract

Cutaneous melanoma refers to a common skin tumor that is dangerous to health with a great risk of metastasis. Previous researches reported that autophagy is associated with the progression of cutaneous melanoma. Nevertheless, the role played by genes with a relation to autophagy (ARG) in the prediction of the course of metastatic cutaneous melanoma is still largely unknown. We observed that thirteen ARGs showed relations to overall survival (OS) in the Cox regression investigation based on a single variate. We developed 2-gene signature, which stratified metastatic cutaneous melanoma cases to groups at great and small risks. Cases suffering from metastatic cutaneous melanoma in the group at great risks had power OS compared with cases at small risks. The risk score, T phase, N phase, and age were proved to be individual factors in terms of the prediction of OS. Besides, the risk scores identified by the two ARGs were significantly correlated with metastatic cutaneous melanoma. Receiver operating characteristic (ROC) curve analysis demonstrated accurate predicting performance exhibited by the 2-gene signature. We also found that the immunization and stromal scores achieved by the group based on large risks were higher compared with those achieved by the group based on small risks. The metastatic cutaneous melanoma cases achieving the score based on small risks acquired greater expression of immune checkpoint molecules as compared with the high-risk group. In conclusion, the 2-ARG gene signature indicated a novel prognostic indicator for prognosis prediction of metastatic cutaneous melanoma, which served as an important tool for guiding the clinical treatment of cutaneous melanoma.

## 1. Introduction

Cutaneous melanoma refers to one type of skin malignant tumor exhibiting high malignancy and ineffective prediction of disease courses [1, 2]. Occult onset and easy invasion and metastasis are important clinical features of cutaneous melanoma [3]. According to the statistics, cutaneous melanoma's incidence in China rose to about 3-5% [4]. Although cutaneous melanoma's incidence within China and other Asian countries is relatively low compared with those in Europe and America, cutaneous melanoma's incidence within China is increasing rapidly [5]. Once cutaneous melanoma cases have distant metastasis, they are diagnosed as advanced or metastatic cm, so the survival time of cutaneous melanoma cases is often short [6]. In the current treatment strategies of metastatic cutaneous melanoma, targeted therapy and immunotherapy play an important role. Metastatic tumor surgery and radiotherapy can also be used selectively [7]. Due to the high metastasis rate of cutaneous melanoma, it is necessary to find a new prognosis model to provide theoretical guidance for the treatment of metastatic cutaneous melanoma.

Autophagy is a process in which cytoplasmic components, or organelles are encapsulated and transported to lysosomes for degradation by forming double membrane autophagosomes [8, 9]. Autophagy can be induced by DNA damage, chemical drugs, ion irradiation, reactive oxygen species, and abnormal growth of tumor cells [10]. According to existing works, autophagy refers to a barrier against malignant transformation of carcinoma cells [11, 12]. Some major oncogenes, such as mTOR and Akt, are considered to be negative regulators of autophagy [13, 14]. According to considerable works, mutant tumor suppressors such as PTEN and TSC1/2 can activate autophagy [15]. It is controversial whether autophagy has a tumor promoting or antitumor effect on the occurrence and development of cancer. At present, according to some studies, autophagy impacts tumor inhibition in the early stage of cancer, but it plays a role in promoting cancer in the formed tumor and contributes to the generation of drug resistance of cancer cells [16, 17]. Therefore, autophagy is considered to help promote the survival of cancer cells in the advanced phase. At present, there are few comprehensive studies on exploring autophagy-relevant genes within the disease course prediction and immunotherapy of metastatic cutaneous melanoma.

Here, we obtained RNA-seq and clinic information regarding cutaneous melanoma cases from The Cancer Genome Atlas (TCGA) database. By several bioinformatics investigations, a multigene signature of ARG was constructed. Relationships of risk model and clinicopathological features of metastatic cutaneous melanoma were confirmed. Then, we carried out Cox regression investigations based on single and multiple variates for the identification of individual factors for the OS of metastatic cutaneous melanoma. A nomogram containing independent prognostic factors was built using “rms” package. We carried out GSVA for exploring the biological processes and pathways involved in the groups based on great and small risks. Furthermore, the analysis was conducted on the landscape of immune infiltration and the expression of immune checkpoint molecules in metastatic cutaneous melanoma. This work might provide a new idea for prognosis and immunotherapy of later phase metastasis melanoma.

## 2. Material and Method

### 2.1. Data Acquisition and Preprocessing

We retrieved the RNA-seq and clinic data regarding the cases with cutaneous melanoma according to The Cancer Genome Atlas (TCGA) cohort (https://portal.gdc.cancer.gov/), which contained 103 primary and 367 metastatic cancer cases. Totally, 232 ARGs were acquired in the Human Autophagy Database (http://autophagy.lu/).

### 2.2. Differentially Expressed Analysis

For screening the gene that achieves different expressions (DEGs) in the metastatic and primary carcinoma samples, the “limma” package was used with regulated *P* value < 0.05 as well as ∣log2 (fold change) | >0.5 [18]. The expression of Top100 genes that achieved different expressions between the metastatic and primary carcinoma samples was shown in a heat map. Then, the DEGs were overlapped with the ARGs to obtain the ARGs that achieved different expressions (DE-ARGs), which were chosen to conduct the subsequent investigation.

### 2.3. Development and Verification of the Prognostic Signature in relation to Autophagy

Subsequently, metastatic cutaneous melanoma cases indiscriminately fell to the test set (*n* = 93) and the training set (*n* = 217) at 3 : 7. To explore whether each DE-ARG is related to overall survival (OS), we performed Cox regression investigation based on a single variate in the training set. The DE-ARGs with the *P* value < 0.05 were identified, followed by the subsequent analysis based on Cox regression investigation based on multiple variates to obtain the best risk model. In the Cox regression investigation based on multiple variates, this study applied the stepwise regression function and set the “direction” as “both.”

Based on the risk model, the score of risk of the respective metastatic cutaneous melanoma case was obtained by: risk score = (*β* 1 *G* 1 + *β*2 *G* 2 + *β*3 *G* 3+⋯+*β* *n* *G* *n*). In the calculated formula, *β* stands the coefficient of gene, and *G* stands the expression level of each gene. The metastatic cutaneous melanoma cases were then stratified into the group based on small risks and group based on large risk in accordance with the mean value of risk score. Furthermore, the OS of these groups was compared using the Kaplan-Meier (K-M) approach on the basis of the log-rank test. In addition, using “survivalROC” R package, we obtained the curves of receiver operating characteristic (ROC) of 1, 3, and 5 years [19]. To be specific, we obtained the area under the curve (AUC) for assessing the risk model's effectiveness. The model of risk was further verified based on the test set.

### 2.4. Functional Enrichment Analysis

According to the gene sets files, we carried out GSVA (PMID: 23323831) for the exploration of the potential biology process and pathways most relevant to groups based on large risk and small risk of metastatic cutaneous melanoma cases. Using the “gsva” package of R software, we carried out a single sample gene set enrichment investigation (ssGSEA) for calculating infiltrating immune cells' proportion in cases with metastatic cutaneous melanoma [20].

### 2.5. Evaluation of Immune Microenvironment

We implemented “ESTIMATE” R package for obtaining the immunization and stromal scores of metastatic cutaneous melanoma cases within TCGA database [21]. Furthermore, the expressions of immunization checkpoint molecules were examined in the metastatic cutaneous melanoma samples.

### 2.6. Statistical Analysis

We carried out the statistical investigations with R software (Version 3.5.3). We investigated various groups' OS on the basis of K-M investigation and compared OS by the log-rank test. Cox regression investigations based on single and multiple variates were applied to investigate the individual prognostic factors for OS. The nomogram containing clinicopathological features was constructed by “rms” package. The differences between two groups were compared using Wilcox.test. Differences were considered statistically significant when *P* < 0.05.

## 3. Result

### 3.1. Identification of Autophagy-Related Genes with Different Expressions (DE-ARGs) in Metastatic Cutaneous Melanoma

**Figure 1:**
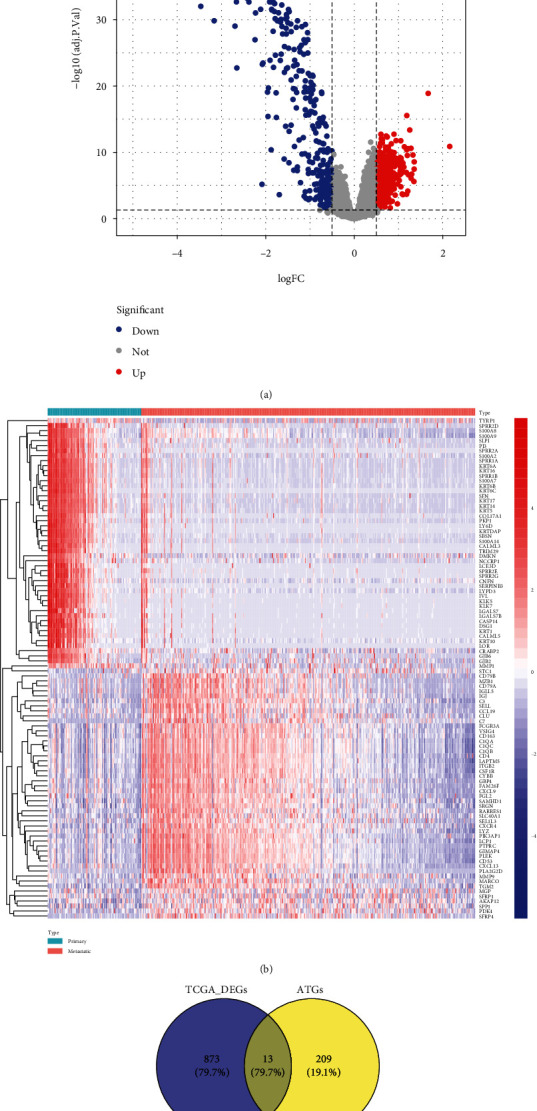
Identification of DE-ARGs in metastatic cutaneous melanoma. (a) Volcano plot of DEGs, including 554 significantly upregulated and 332 significantly downregulated genes were identified in metastatic cancer samples compared to the primary cancer samples. Threshold: ∣log2 (fold change) | >0.5 and adjusted *P* value < 0.05. Blue dot for downregulated genes; red dot for upregulated genes. (b) The expression of Top100 DEGs between the primary and metastatic cancer samples of TCGA database. (c) Venn plot for combination of 886 DEGs (blue circle) and 222 ARGs (yellow circle), obtaining 13 DE-ARGs.

To seek the ARGs in relations to the disease course prediction of cutaneous melanoma, we first analyzed the DEGs between the primary and metastatic cancer samples of TCGA database using “limma” package. Under the threshold of regulated *P* value < 0.05 as well as ∣log2 (fold change) | >0.5, we identified 886 DEGs in total, covering 554 significantly upregulated and 332 significantly decreased genes within metastatic cancer samples in comparison with the primary cancer samples (Figure 1(a)).  Figure 1(b) reveals the expression of Top100 DEGs between the primary and metastatic cancer samples of the TCGA database. Furthermore, we combined the 886 DEGs with 222 ARGs, obtaining 13 DE-ARGs, to carry out the following investigation (Figure 1(c)).

### 3.2. Establishment and Validation of the Prognostic Signature in relation to Autophagy

**Figure 2:**
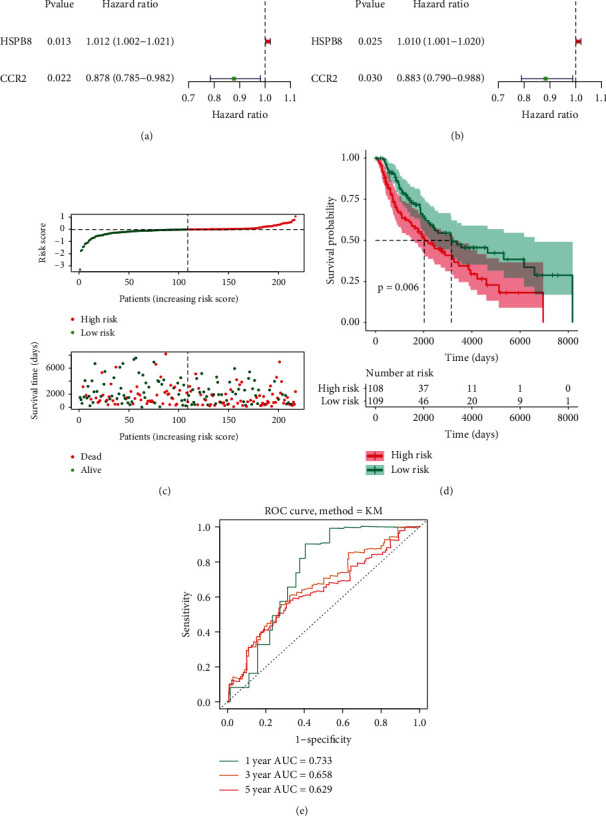
Training set for establishment of the autophagy-related prognostic signature. (a) The univariate Cox regression analysis for HSPB8, CCR2 with the survival of metastatic cutaneous melanoma in the training set. (b) The multivariate Cox regression analysis for combination of HSPB8 and CCR2 with the survival of metastatic cutaneous melanoma in the training set. (c) The distribution of patient risk scores and survival status in the training set. (d) The K-M survival analysis of the metastatic cutaneous melanoma patients between high-risk score and low-risk patients in overall survival. Red line for high risk; green line for low risk. (e) The time-dependent ROC curve and AUC values of 1-, 3-, and 5-year OS in the training set.

**Figure 3:**
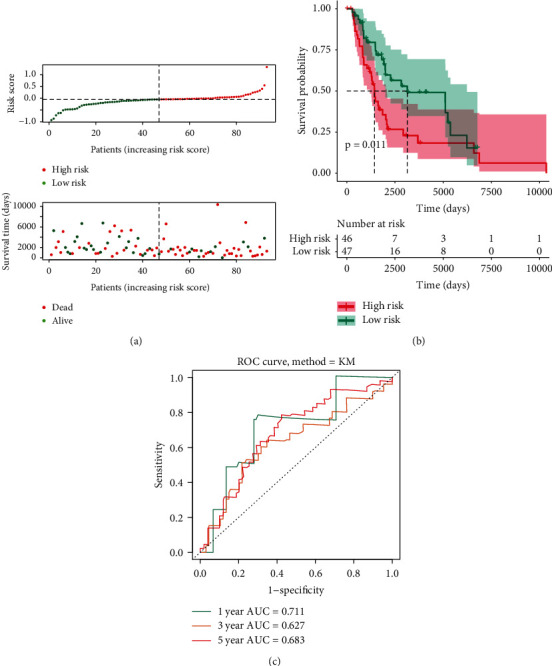
Validation set for the autophagy-related prognostic signature. (a) The distribution of patient risk scores and survival status in the validation set. (b) The K-M survival analysis of the metastatic cutaneous melanoma patients between high-risk score and low-risk patients in overall survival. Red line for high risk; green line for low risk. (c) The time-dependent ROC curve and AUC values of 1-, 3-, and 5-year OS in the validation set.

For more specifically assessing whether the DE-ARGs are related to the survival of metastatic cutaneous melanoma cases, the Cox regression investigation based on single variate was performed within the training set (Figure 2(a)). The result indicated that two genes had significant relations to the metastatic cutaneous melanoma cases' OS (*P* < 0.05), of which HSPB8 was a risk factor (HR > 1), and CCR2 was a protective factor (HR < 1) in metastatic cutaneous melanoma. Then, we reported a 2-gene signature based on the Cox regression investigation based on multiple variates (Figure 2(b)). Two ARGs and their corresponding coefficients were utilized for determining the score of risk of the respective metastatic cutaneous melanoma case. The calculated equation in terms of the score of risk is presented as 0.0103 × (expression value of HSPB8) + (−0.124) × (expression value of CCR2) within the training and test sets.  Figure 2(c) illustrates the distributions of case risk scores and survivals. The K-M survival investigation revealed that the metastatic cutaneous melanoma cases with high-risk scores had a significantly poorer OS in comparison with that of cases based on small risks (Figure 2(c), *P* < 0.05). Besides, according to the ROC depending on time, the AUC of 1-, 3-, and 5-year OS reached 0.733, 0.658, and 0.629, separately (Figure 2(e)). Lastly, we verified the 2-ARGs prognosis signature with the use of OS information according to the test set, complying with the results of the training set (Figures 3(a)–3(c)), in which AUC of 1-, 3-, and 5-year OS reached 0.711, 0.627, and 0.683, separately. Taken together, all the results suggested the reliable predicting performance exhibited by the prognosis signature constructed by the two ARGs.

### 3.3. The Associations between the Risk Score and the Clinicopathological Features in Cases with Metastatic Cutaneous Melanoma

**Figure 4:**
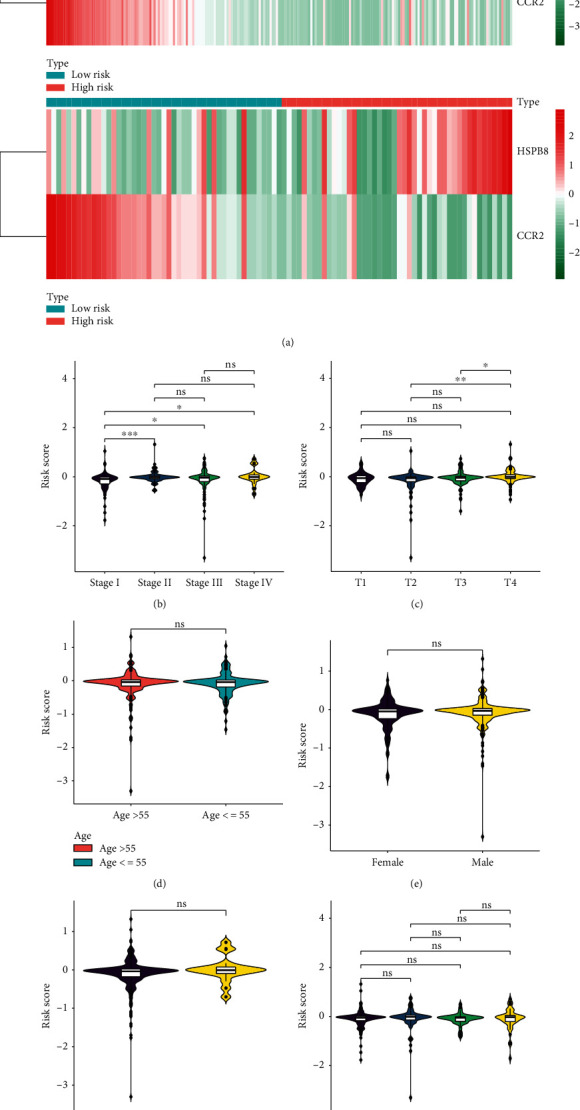
The associations between the risk score and the clinicopathological features in patients with metastatic cutaneous melanoma. (a) The expression of the two screened ARGs between the low- and high-risk samples in the TCGA dataset are displayed by heatmaps. Left: training set; right: validation set. Red for high risk; blue line for Low risk. (b)–(g) Violin plot for the associations between the risk score and clinicopathological features, involved with stage and T stage, age, gender, M, and N stage, respectively. ns: no significance; ∗: *P* < 0.05; ∗∗: *P* < 0.01; ∗∗∗: *P* < 0.001.

The expression of the two screened ARGs of the small- and great-risk samples within the TCGA dataset is illustrated by heatmaps (Figure 4(a)). We observed differences with statistical significance in these groups within the training and test sets. To further investigate the associations of the risk scores and clinicopathology characteristics, this study quantitatively analyzed the risk score in metastatic cutaneous melanoma (Figures 4(b)–4(g)). As a result, the risk scores and the survival probability were both significantly different in these groups with the TCGA dataset compartmentalized by Phase and T phase. However, the risk scores were no significant differences in these groups divided by age, gender, M, and N phase. Moreover, the survival probability was significantly different in these groups classified by age and N phase.

**Figure 5:**
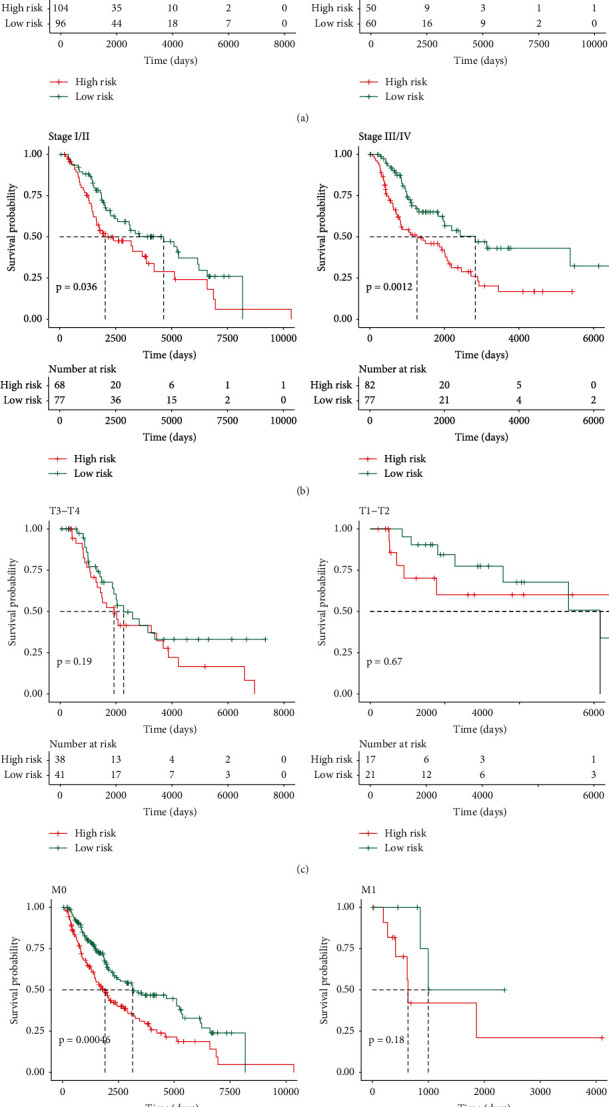
The survival analysis between the risk score and the stratified clinicopathological features in patients with metastatic cutaneous melanoma. (a)–(d) The K-M survival analysis of the metastatic cutaneous melanoma patients between high-risk score had and low-risk patients in overall survival, involved with stage I-II, stage III-IV, M0, M1, N0, and N1, respectively; red line for high risk; green line for low risk.

By the same taken, we performed the stratified survival investigation on the clinicopathological features. According to Figure 5(a), greater risk scores showed relations to lower survival according to male cases, whereas female cases showed an insignificant difference. Furthermore, high-risk score noticeably caused a poorer OS in metastatic cutaneous melanoma cases with Phase I-II, Phase III-IV (Figure 5(b)), M0, N0, and N1, whereas it was not found to be risk factors in terms of metastatic cutaneous melanoma cases with phase M1 (Figures 5(c) and 5(d)). The results demonstrated that the risk scores identified by two ARGs were significantly correlated with metastatic cutaneous melanoma.

### 3.4. Individual Prognosis Value Achieved by the 2-Gene Signature within Metastatic Cutaneous Melanoma

**Figure 6:**
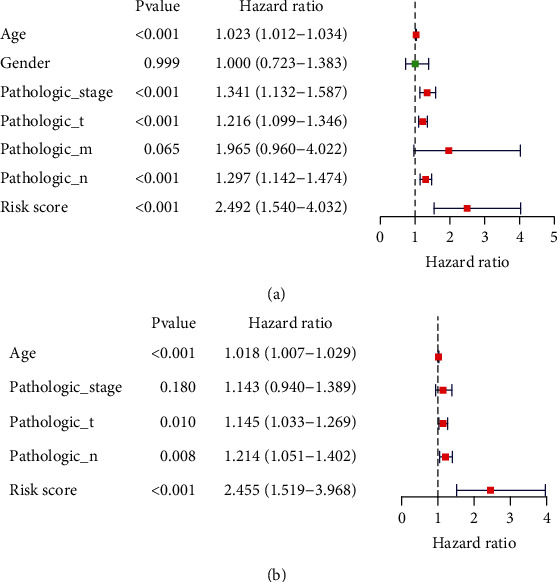
Independent prognostic value of the 2-gene signature in metastatic cutaneous melanoma. (a) The result of univariate Cox regression between the risk score and clinicopathological features of age, gender, T stage, N stage, M stage, and pathological stage. (b) The result of multivariate Cox regression between the risk score and clinicopathological features of age, gender, T stage, N stage, M stage, and pathological stage.

To further demonstrate whether this risk score acted as an individual factor in terms of the prediction of the course of metastatic cutaneous melanoma according to the clinicopathology characteristics of age, gender, T phase, N phase, M phase, and Pathological Phase. The result of univariate Cox regression investigations indicated that the risk score, age, pathologic phase, T phase, and N phase showed noticeable relations to the prediction of the course of metastatic cutaneous melanoma (Figure 6(b), *P* < 0.05). Based on these significant clinicopathological features, we further performed Cox regression investigation based on multiple variates. As revealed from the result, the risk score, T phase, N phase, and age are significantly correlated with the OS (Figure 6(b), *P* < 0.05).

### 3.5. Development and Evaluation of the Nomogram for OS in Metastatic Cutaneous Melanoma

**Figure 7:**
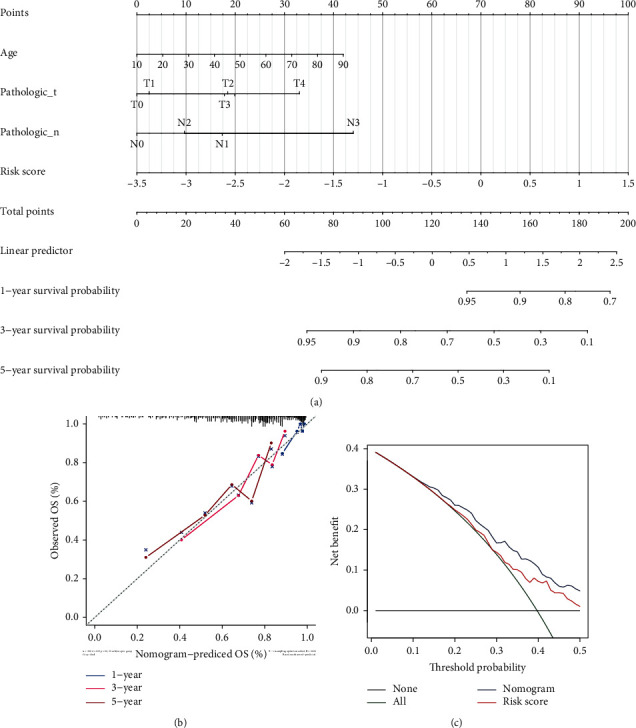
Clinical prediction model of clinicopathological features for OS in metastatic cutaneous melanoma. (a) The nomogram plot using the five clinicopathological features including risk score, age, pathologic stage, T stage, and N stage to predict the OS. (b) The calibration curve for accurate prediction efficiency of 1-, 3-, and 5- year survival in the TCGA database. (c) The decision curve analysis (DCA) compared the risk model with the addition of clinicopathological features by net benefit.

Then, we established the nomogram using the five clinicopathological features including risk score, age, pathologic phase, T phase, and N phase (Figure 7(a)). The accurate prediction efficiency of 1-year survival and 3-year survival in the TCGA database was investigated by the calibration curve (Figure 7(b)). Moreover, according to the analysis in terms of decision curve (DCA), the risk model with the addition of clinicopathological features showed better net benefit than the risk only model (Figure 7(c)), which suggested the ability of the nomogram in the accurate prediction of the prognosis of metastatic cutaneous melanoma cases.

### 3.6. Functional Analyses in the TCGA Database

**Figure 8:**
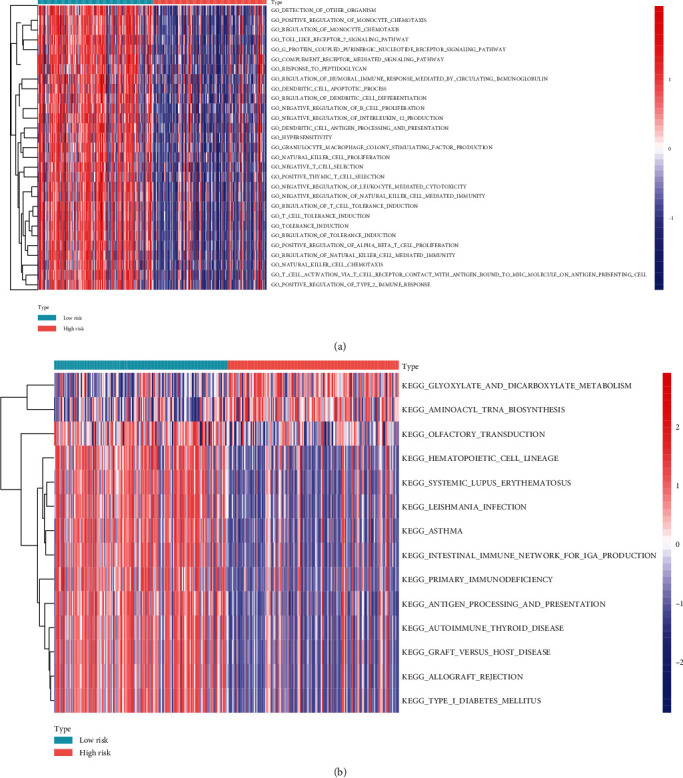
The result of functional analyses in the TCGA database. (a) GSVA analysis to elucidate the biological processes and pathways related to the risk score for GO Terms. (b) GSVA analysis to elucidate the biological processes and pathways related to the risk score for KEGG terms.

Furthermore, GSVA was performed for elucidating the biology process and channels related to the risk score. According to Figure 8(a), many immune-related GO terms, including GO_TOLL_LIKE_RECEPTOR_7_SIGNALING_PATHWAY, GO_REGULATION_OF_NATURAL_KILLER_CELL_MEDIATED_IMMUNITY, GO_NATURAL_KILLER_CELL_CHEMOTAXIS, GO_T_CELL_ACTIVATION_VIA_T_CELL_RECEPTOR_CONTACT_WITH_ANTIGEN_BOUND_TO_MHC_MOLECULE_ON_ANTIGEN_PRESENTING_CELL, and GO_POSITIVE_REGULATION_OF_TYPE_2_IMMUNE_RESPONSE, showed enrichment within the groups with the score based on small risks. Besides, the KEGG pathway analyses also indicated the KEGG_INTESTINAL_IMMUNE_NETWORK_FOR_IGA_PRODUCTION, KEGG_PRIMARY_IMMUNODEFICIENCY, KEGG_ANTIGEN_PROCESSING_AND_PRESENTATION, KEGG_AUTOIMMUNE_THYROID_DISEASE, KEGG_GRAFT_VERSUS_HOST_DISEASE, KEGG_ALLOGRAFT_REJECTION, and KEGG_TYPE_I_DIABETES_MELLITUS were enriched in high-risk groups (Figure 8(b)), further suggesting that these prognostic genes might participate in the progression of cutaneous melanoma metastasis.

### 3.7. The Landscape of Immune Infiltration within Metastatic Cutaneous Melanoma

**Figure 9:**
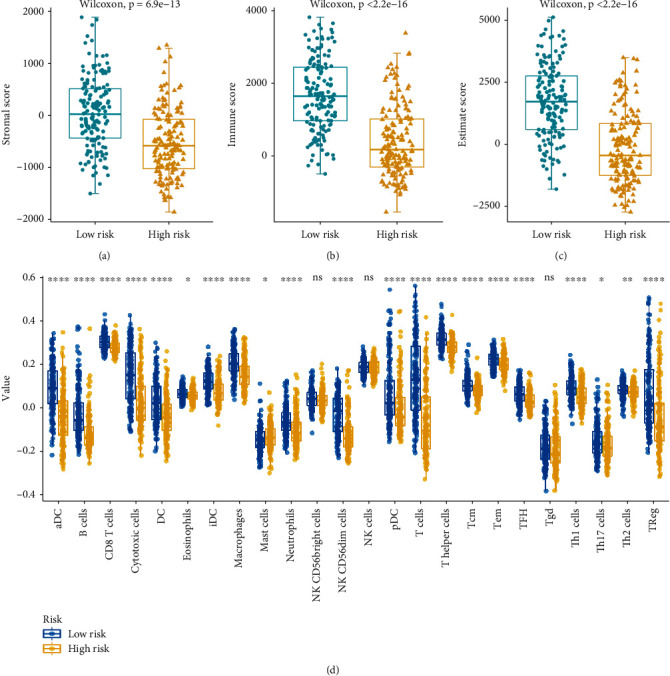
The landscape of immune infiltration in metastatic cutaneous melanoma. (a) ESTIMATE algorithm result of stromal score in metastatic cutaneous melanoma tumor between low risk and high risk. (b) ESTIMATE algorithm result of immune score in metastatic cutaneous melanoma tumor between low risk and high risk. (c) ESTIMATE score in metastatic cutaneous melanoma tumor between low risk and high risk. (d) The correlation between the risk score and immune status, aDC, B cells, CD8 T cells, cytotoxic cells, DC, eosinophils, iDC, macrophages, mast cells, neutrophils, NK CD56dim cells, pDC, T cells, T helper cells, Tcm, Tem, TFH, Th1 cells, Th17 cells, Th2 cells, and Treg between the low-risk and high-risk groups in the TCGA cohort. ns: no significance; ∗: *P* < 0.05; ∗∗: *P* < 0.01; ∗∗∗: *P* < 0.001.

With the use of the ESTIMATE, the content of stromal and immunization cells within metastatic cutaneous melanoma tumor tissues was calculated. We found that the immunization and stromal scores achieved by the group based on great risks exceeded those achieved by the group based on low risks (Figures 9(a) and 9(b), *P* < 0.05). Moreover, we analyzed the ESTIMATE of the two groups and obtained the same trends (Figure 9(c), *P* < 0.05). To investigate relations of the score of risk and immunization state, this study determined the enrichment score of immunization gene sets. Interestingly, the score of Th1 cells, TFH, Tem, Tcm, T helper cells, T cells, pDC NK CD56dim cells, neutrophils, mast cells, macrophages, iDC, iDC, eosinophils, DC, cytotoxic cells, CD8 T cells, B cells, aDC, Th17 cells, Th2 cells, and TReg showed noticeable distinctions in the groups based on small and great risks in the TCGA group (all *P* < 0.05, Figure 9(d)). Accordingly, the immune infiltration in metastatic cutaneous melanoma may act as targets for immunotherapy and may have potential clinical implications.

### 3.8. The Expressions of Immune Checkpoint Molecules

**Figure 10:**
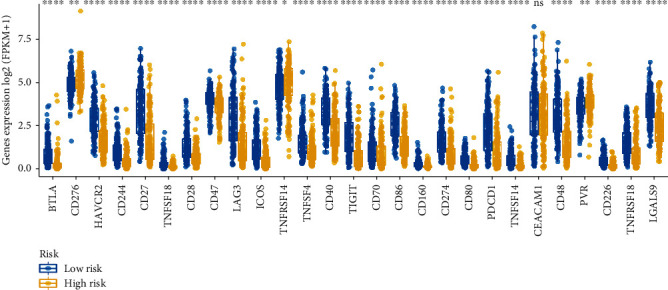
The expression of immune checkpoint molecules and the correlation between immune checkpoint molecules and risk score in the TCGA cohort. ns: no significance; ∗: *P* < 0.05; ∗∗: *P* < 0.01; ∗∗∗: *P* < 0.001.

Programmed cell death receptor ligand 1 (PD-L1) and blocking programmed cell death 1 receptor (PD-1) have been a special interest in developing antibodies for a subset of cancer cases (PMID: 31488176). Therefore, immune checkpoint proteins have diverse clinical implications in the immunotherapy of cancers. We then investigated any potential relation of the score of risk and the expressions achieved by immunization checkpoint molecules. According to Figure 10, the metastatic cutaneous melanoma cases achieving low-risk score had greater expression of immune checkpoint molecules than the high-risk group. Accordingly, the low-risk cases suffering from metastatic cutaneous melanoma might have a more promising treatment to respond for immunotherapies.

## 4. Discussion

Autophagy and autophagy-related genes play an important role in metastatic cutaneous melanoma. Ryabaya et al. reported that autophagy inhibitor integration chloroquine or LY294002 and TMZ could enhance the cytotoxicity of alkylating agents on human melanoma cell lines [22]. Zhang et al. investigated that CX-F9, a novel Ribosomal S6 Kinase 2 (RSK2) inhibitor, could significantly suppress the proliferation, invasion, and autophagy of melanoma in vitro and in vivo [23]. In the present study, thirteen ARGs showed correlations to OS in the Cox regression investigation based on a single variate. A 2-gene signature was developed, which stratified metastatic cutaneous melanoma cases into the groups based on great and small risks. Cases with metastatic cutaneous melanoma in the high-risk group had worse OS than that of the group based on small risks. The risk score, T phase, N phase, and age were proved to be individual factors for predicting OS. Besides, the risk scores identified by the two ARGs were significantly correlated with metastatic cutaneous melanoma. Receiver operating characteristic (ROC) curve analysis demonstrated accurate predictive performance of the 2-gene signature. Functional enrichment analysis indicated that immune-related biological processes and channels were significantly enriched. The infiltrating immune cell content was different between the two risk groups. We also found that the immune scores and stromal scores of the high-risk group were higher compared with that of group based on low risks. The metastatic cutaneous melanoma cases achieving low-risk scores had greater expression of immune checkpoint molecules as compared with the high-risk group.

Although autophagy-related genes play a crucial role in some diseases, there is no comprehensive study on the prognosis and immunotherapy of autophagy-related genes in cases suffering from metastatic cutaneous melanoma. This study reports for the first time the role of autophagy-related genes in the prognosis and immunotherapy of cases with metastatic cutaneous melanoma. The risk models of HspB8 and CCR2 were established by univariate Cox and multivariate Cox analysis. HspB8 acts as an oncogene in several cancers. Shen et al. reported that HSPB8 promoted cancer cell growth by activating the ERK-CREB pathway and predicted a poor prognosis in gastric cancer cases [24]. The expression of HSPB8 is investigated to correlate with breast cancer progression [25]. In addition, HSPB8 is responsible for the rug resistance of breast cancer cells. The mTOR inhibitor (AZD8055) could inhibit the tamoxifen resistance in breast cancer cells by suppressing the expression of HSPB8 [26]. Several studies have shown that CCR2 acts as a novel biomarker in metastatic cutaneous melanoma [27, 28]. Furthermore, Zhang et al. demonstrated that Toll-like receptors 7 and 8 expression correlated with the expression of immune biomarkers (CCR2, CCR5, CCL3, and CCL5) and positively predicted the clinical outcome of cases with melanoma [29]. This work is the first time to stratify cases with metastatic cutaneous melanoma based on autophagy-related genes, which provides new insights for predicting the efficacy of immunotherapy and possible differentiation targets.

We further performed functional analyses of ARGs in the TCGA database. The results of GSVA elucidated that autophagy-related genes may be closely related to tumor immunity. For example, the ARGs were enriched in GO_TOLL_LIKE_RECEPTOR_7_SIGNALING_PATHWAY, GO_NATURAL_KILLER_CELL_CHEMOTAXIS, and GO_POSITIVE_REGULATION_OF_TYPE_2_IMMUNE_RESPONSE. These pathways are associated with the tumor immunity of melanoma [30–32]. The KEGG pathway analyses also indicated the KEGG_INTESTINAL_IMMUNE_NETWORK_FOR_IGA_PRODUCTION, KEGG_PRIMARY_IMMUNODEFICIENCY, KEGG_ANTIGEN_PROCESSING_AND_PRESENTATION, KEGG_AUTOIMMUNE_THYROID_DISEASE, KEGG_GRAFT_VERSUS_HOST_DISEASE, KEGG_ALLOGRAFT_REJECTION, and KEGG_TYPE_I_DIABETES_MELLITUS were enriched in high-risk groups. These pathways participated in the process of immune escape in cutaneous melanoma metastasis [33, 34]. The results revealed that these prognostic genes might participate in the progression of cutaneous melanoma metastasis.

Since the discovery of immune checkpoint proteins, the development of antibodies against programmed cell death receptor-1 (PD-1) and programmed cell death receptor ligand-1 (PD-L1) has aroused special interest in the treatment of some cancer cases [35, 36]. PD-1 signal carries out the negative regulation of T cell-mediated immune response, which is one of the mechanisms of tumor escaping antigen-specific T cell immune response [37]. It facilitates tumor development and progression by improving the survival rate of tumor cells [38]. In this context, PD-1 signaling is a valuable new and effective target for cancer immunotherapy. Javed et al. reported that significant differences existed in PD-L1 expression between metastatic uveal melanoma and metastatic cutaneous melanoma. The higher PD-L1 expression was observed in metastatic cutaneous melanoma [39]. This work reported that the expressions achieved by the mentioned key immune checkpoints increased in the group based on small risks. The key immune checkpoints including BTLA, CD86, CD244, and PDCD1 are recognized as predictors of sentinel lymph node metastasis in cutaneous melanoma [40, 41]. Our results indicated that the low-risk cases with metastatic cutaneous melanoma might have a more promising treatment to respond for immunotherapies.

In conclusion, the 2-ARG gene signature indicates a novel prognostic indicator for prognosis prediction of metastatic cutaneous melanoma, which served as an important tool for guiding the clinical treatment of cutaneous melanoma.

## Data Availability

The datasets used and analyzed during the current study are available from the corresponding author on reasonable request.

## References

[1] Hartman R. I., Lin J. Y. (2019). Cutaneous Melanoma--A Review in Detection, Staging, and Management. *Hematology/Oncology Clinics of North America*.

[2] Coricovac D., Dehelean C., Moaca E. A. (2018). Cutaneous melanoma-a long road from experimental models to clinical outcome: a review. *International Journal of Molecular Sciences*.

[3] Read J., Wadt K. A., Hayward N. K. (2016). Melanoma genetics. *Journal of Medical Genetics*.

[4] Nassar K. W., Tan A. C. (2020). The mutational landscape of mucosal melanoma. *Seminars in Cancer Biology*.

[5] Tang B., Chi Z., Guo J. (2020). Toripalimab for the treatment of melanoma. *Expert Opinion on Biological Therapy*.

[6] Gorayski P., Burmeister B., Foote M. (2015). Radiotherapy for cutaneous melanoma: current and future applications. *Future Oncology*.

[7] Weiss S. A., Hanniford D., Hernando E., Osman I. (2015). Revisiting determinants of prognosis in cutaneous melanoma. *Cancer*.

[8] Mizushima N., Komatsu M. (2011). Autophagy: renovation of cells and tissues. *Cell*.

[9] Nazio F., Bordi M., Cianfanelli V., Locatelli F., Cecconi F. (2019). Autophagy and cancer stem cells: molecular mechanisms and therapeutic applications. *Cell Death and Differentiation*.

[10] Levine B., Kroemer G. (2019). Biological functions of autophagy genes: a disease perspective. *Cell*.

[11] Onorati A. V., Dyczynski M., Ojha R., Amaravadi R. K. (2018). Targeting autophagy in cancer. *Cancer*.

[12] Towers C. G., Fitzwalter B. E., Regan D. (2019). Cancer cells upregulate NRF2 signaling to adapt to autophagy inhibition. *Developmental Cell*.

[13] Cheng Z. (2019). The FoxO-autophagy axis in health and disease. *Trends in Endocrinology and Metabolism*.

[14] Boya P., Reggiori F., Codogno P. (2013). Emerging regulation and functions of autophagy. *Nature Cell Biology*.

[15] Xiang H., Zhang J., Lin C., Zhang L., Liu B., Ouyang L. (2020). Targeting autophagy-related protein kinases for potential therapeutic purpose. *Acta Pharmaceutica Sinica B*.

[16] Amaravadi R. K., Kimmelman A. C., Debnath J. (2019). Targeting autophagy in cancer: recent advances and future directions. *Cancer Discovery*.

[17] Glick D., Barth S., Macleod K. F. (2010). Autophagy: cellular and molecular mechanisms. *The Journal of Pathology*.

[18] Ritchie M. E., Phipson B., Wu D. (2015). Limma powers differential expression analyses for RNA-sequencing and microarray studies. *Nucleic Acids Research*.

[19] Lin P., He R. Q., Ma F. C. (2018). Systematic analysis of survival-associated alternative splicing signatures in gastrointestinal pan-adenocarcinomas. *eBioMedicine*.

[20] Hänzelmann S., Castelo R., Guinney J. (2013). GSVA: gene set variation analysis for microarray and RNA-seq data. *BMC Bioinformatics*.

[21] Yavorska O. O., Burgess S. (2017). MendelianRandomization: an R package for performing Mendelian randomization analyses using summarized data. *International Journal of Epidemiology*.

[22] Ryabaya O. O., Inshakov A. N., Egorova A. V. (2017). Autophagy inhibitors chloroquine and LY294002 enhance temozolomide cytotoxicity on cutaneous melanoma cell lines in vitro. *Anti-Cancer Drugs*.

[23] Zhang X., Cai L., Zhao S. (2019). CX-F9, a novel RSK2 inhibitor, suppresses cutaneous melanoma cells proliferation and metastasis through regulating autophagy. *Biochemical Pharmacology*.

[24] Shen J., Li M., Min L. (2018). HSPB8 promotes cancer cell growth by activating the ERK-CREB pathway and is indicative of a poor prognosis in gastric cancer patients. *Oncology Reports*.

[25] Shi J. J., Chen S. M., Guo C. L., Li Y. X., Ding J., Meng L. H. (2018). The mTOR inhibitor AZD8055 overcomes tamoxifen resistance in breast cancer cells by down-regulating HSPB8. *Acta Pharmacologica Sinica*.

[26] Chen S. M., Guo C. L., Shi J. J. (2014). HSP90 inhibitor AUY922 abrogates up-regulation of RTKs by mTOR inhibitor AZD8055 and potentiates its antiproliferative activity in human breast cancer. *International Journal of Cancer*.

[27] Tu M. M., Abdel-Hafiz H. A., Jones R. T. (2020). Inhibition of the CCL2 receptor, CCR2, enhances tumor response to immune checkpoint therapy. *Commun Biol*.

[28] Ngiow S. F., Knight D. A., Ribas A., McArthur G. A., Smyth M. J. (2013). BRAF-targeted therapy and immune responses to melanoma. *Oncoimmunology*.

[29] Zhang M., Yan Z., Wang J., Yao X. (2017). Toll-like receptors 7 and 8 expression correlates with the expression of immune biomarkers and positively predicts the clinical outcome of patients with melanoma. *Oncotargets and Therapy*.

[30] Rathore M., Girard C., Ohanna M. (2019). Cancer cell-derived long pentraxin 3 (PTX3) promotes melanoma migration through a toll-like receptor 4 (TLR4)/NF-*κ*B signaling pathway. *Oncogene*.

[31] Kim J., Kim J. S., Lee H. K. (2018). CXCR3-deficient natural killer cells fail to migrate to B16F10 melanoma cells. *International Immunopharmacology*.

[32] Fang R., Hara H., Sakai S. (2014). Type I interferon signaling regulates activation of the absent in melanoma 2 inflammasome during Streptococcus pneumoniae infection. *Infection and Immunity*.

[33] Huang L., Chen H., Xu Y., Chen J., Liu Z., Xu Q. (2020). Correlation of tumor-infiltrating immune cells of melanoma with overall survival by immunogenomic analysis. *Cancer Medicine*.

[34] Kamińska-Winciorek G., Cybulska-Stopa B., Lugowska I., Ziobro M., Rutkowski P. (2019). Principles of prophylactic and therapeutic management of skin toxicity during treatment with checkpoint inhibitors. *Advances in Dermatology and Allergology/Postȩpy Dermatologii i Alergologii*.

[35] Schütz F., Stefanovic S., Mayer L., von Au A., Domschke C., Sohn C. (2017). PD-1/PD-L1 pathway in breast cancer. *Oncology Research and Treatment*.

[36] Dermani F. K., Samadi P., Rahmani G., Kohlan A. K., Najafi R. (2019). PD-1/PD-L1 immune checkpoint: potential target for cancer therapy. *Journal of Cellular Physiology*.

[37] Cha J. H., Chan L. C., Li C. W., Hsu J. L., Hung M. C. (2019). Mechanisms controlling PD-L1 expression in cancer. *Molecular Cell*.

[38] Rotte A. (2019). Combination of CTLA-4 and PD-1 blockers for treatment of cancer. *Journal of Experimental & Clinical Cancer Research*.

[39] Javed A., Arguello D., Johnston C. (2017). PD-L1 expression in tumor metastasis is different between uveal melanoma and cutaneous melanoma. *Immunotherapy*.

[40] Barrios D. M., Do M. H., Phillips G. S. (2020). Immune checkpoint inhibitors to treat cutaneous malignancies. *Journal of the American Academy of Dermatology*.

[41] Sahin U., Oehm P., Derhovanessian E. (2020). An RNA vaccine drives immunity in checkpoint-inhibitor-treated melanoma. *Nature*.

